# Gentisic Acid Stimulates Keratinocyte Proliferation through ERK1/2 Phosphorylation

**DOI:** 10.7150/ijms.36484

**Published:** 2020-02-24

**Authors:** Minho Kim, JaeGoo Kim, Yu-Kyong Shin, Ki-Young Kim

**Affiliations:** 1 Graduate School of Biotechnology, Kyung Hee University, Yongin-si, Gyeonggi-do, Republic of Korea; 2 College of Life Science, Kyung Hee University, Yongin-si, Gyeonggi-do, Republic of Korea

**Keywords:** HaCaT cell, MAP kinase pathway, wound healing, re-epithelialization

## Abstract

Keratinocyte proliferation is important for skin wound healing. The wound healing process includes blood clotting around the wound, removal of dead cells and pathogens through inflammation, and then re-epithelialization through proliferation and maturation. Proliferation assay was performed on acid natural compounds to identify candidates for natural-derived components of skin injury treatment. We found that gentisic acid promoted high cell proliferation activity compared with other compounds. Gentisic acid improved HaCaT cell proliferation by over 20% in MTT assay. Gentisic acid also had higher healing activity in an *in vitro* wound healing assay than allantoin as a positive control. Furthermore, we have identified how the treatment of gentisic acid can increase proliferation in the cell. Western blot analysis of proteins in the mitogen-activated protein (MAP) kinase signaling pathway showed that ERK1/2 phosphorylation was increased by gentisic acid treatment. Thus, our study indicates that gentisic acid promotes the proliferation of keratinocyte by phosphorylation of ERK1/2.

## Introduction

Skin wound healing is one mechanism that maintains homeostasis in organisms. In general, the wound healing process is classified time-dependently into 4 phases: coagulation and haemostasis phase, inflammatory phase, proliferative phase and remodeling phase 1. During the proliferative stage, recovery, and restoration of the basal keratinocyte layer in the basement membrane between the epidermis and the dermis begin actively proliferating through various signaling molecules. When a certain level of repair is achieved, the cytoplasmic shape of the keratinocyte is altered to move to the upper layer of the epidermis, differentiate, and transform through different cell layers to reach their final maturation stage. Thus, proliferation and migration of keratinocytes suture the wound site during wound healing 2, 3.

The MAP kinase signaling pathway is important for integrating external signals from mitogens, such as epidermal growth factor (EGF), into signaling events promoting cell growth and proliferation in many mammalian cell types 4, 5. The MAP kinase signaling pathway is composed of signaling receptors and kinase cascades 6. In the kinase cascades, ERK1/2, JNK, and P-38 kinases are involved in cell cycle regulation and proliferation. In the presence of an extracellular signal, for instance, growth factors, the signal is transmitted in order through MAPKKK, MAPKK, MAPK, and then the substrates 7, 8.

In the ERK1/2 pathway, signaling is transmitted in order through Raf, MEK1/2, and ERK1/2. As a result, phosphorylated ERK1/2 influences gene expression, which contributes to the regulation of cell proliferation 9-12. For example, in alkali burn wound recovery, phosphorylation of ERK1/2 has been promoted by the compound body protective compound-157 in human umbilical vein endothelial cells to facilitate proliferation and wound repair 13. This ERK1/2 pathway is also important in keratinocyte proliferation 13.

**Figure 1:**
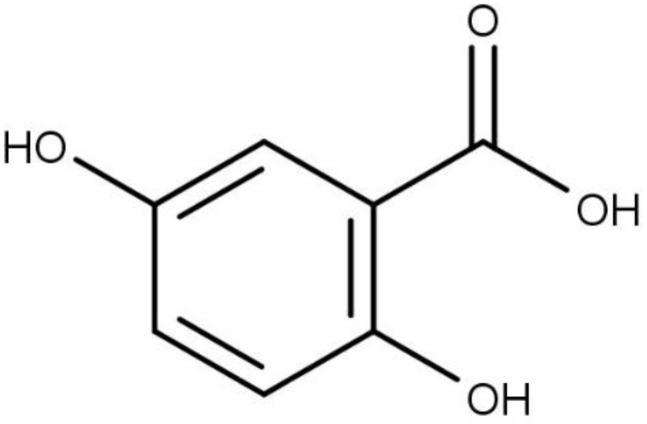
2D structure of gentisic acid.

Gentisic acid (2-(2,5-dihydroxyphenyl)acetic acid) (Figure 1) is a quinonoid phenolic acid synthesized in plants. Genstisic acid has one carbonyl group and two hydroxyl groups based on one benzene ring. One hydroxyl group (near the carbonyl group) can form an intramolecular hydrogen bond with the carbonyl group. This structure causes the formation of gentisinate, resulting in a more acidic of the carboxyl groups. The hydroxyl groups on the other side which cannot interact with any intra-molecules can freely react with other molecules 15.

Like sialic acid in plants, gentisic acid has immune activity against viral plant pathogens. Mammals, including humans, obtain gentisic acid by ingesting fruits and vegetables, and gentisic acid is also produced by the metabolic breakdown of aspirin 16, 17.

Gentisic acid has some biological effects, including the inhibition of angiogenesis as an FGF competitive inhibitor 18, skin whitening through melanocyte tyrosinase inhibition 19, and inhibition of low-density lipoprotein oxidation and cholesterol ester hydroperoxide formation 20. Besides, gentisic acid can be extracted from the African tree *Alchornea cordifolia* 21 and its extract is effective in wound healing 22.

In this study, our purpose was to find an effective natural compound for wound healing, especially in the epithelial wounds, by promoting the proliferation of keratinocyte. We screened acidic natural compounds to find natural compounds that are effective in healing wounds. Among them, gentisic acid was the most potent compound in a cell proliferation assay. Re-epithelialization of gentisic acid was better than allantoin, which is already known to be effective 23. Besides, we confirmed the effect of gentisic acid on keratinocytes in terms of cell signaling pathway by western blot analysis, indicates that the proliferation of keratinocytes is increased by promoting phosphorylation of ERK 1/2.

## Materials and Methods

### Natural compounds

Gentisic acid (Figure 1), allantoin (positive control), cis-8,11,14-Eicosatrienoic acid, Linoleic acid, and γ-Linolenic acid were purchased from Sigma Aldrich (St. Louis, MO, USA, Catalog no. 149357, A0349000, E4504, L2376, L2378) 23, 24. Other natural compounds were purchased from ChemFaces (Wuhan, China, Catalog no. Esculentic acid: CFN99059, Gallic acid: CFN99624, Salvianolic acid A: CFN99161, Actinidic acid: CFN98444, Pimaric acid: CFN99382, Corosolic acid: CFN98685, Caffeic acid: CFN99190, Loganic acid: CFN98212). Compounds were dissolved in dimethyl sulfoxide (DMSO) and stored at -20°C (10mg/ml).

### Cell lineage and culture conditions

The human keratinocyte cell line HaCaT (HaCaT cell was kindly provided by COSMAX BIO, Jecheon, Korea) and human skin fibroblast cell line CCD-986sk (CCD-986sk was purchased from the Korean cell line, Seoul, Korea) was cultured in Dulbecco's Modified Eagle's Medium containing 10% Fetal Bovine Serum and 1% penicillin-streptomycin at 37°C in a 5% CO_2_ atmosphere 25.

### Cell proliferation assay

Cell proliferation assays were performed with the compounds in Table 1 to find the most effective among wounded natural acid compounds. This assay is based on the MTT assay 24, 25. HaCaT cells (10^3^ cells per well) and CCD-986sk (5×10^4^ cells per well) were seeded into a 96-well plate 26. The medium was exchanged with a serum-free medium containing various concentrations of gentisic acid (0, 1, 5, 10, 50, and 100 μg/mL) and incubate 24h. MTT (3-(4,5-dimethyl-thiazol-2-yl)-2,5-diphenyltetrazolium bromide, Sigma) dissolved in phosphate-buffered saline (PBS) was added to each well (final concentration of 0.5 mg/ml) and incubated for 3 hours. The medium was removed and cells were suspended in 100μl of DMSO for 10 minutes. The cell proliferation rate was calculated from optical density (OD_540_) values measured using a microplate reader (BioTek Instruments, Korea). The data was presented as a percentage of the control. The experiments were independently repeated three times 27, 28, 29, 30.

### *In vitro* wound healing assay

HaCaT cells (3×10^5^ cells per well) were seeded into 6-well plates and cultured to a nearly confluent, cell monolayer (90-100%). Using a 20-200μl pipet tip, a parallel linear wound was generated in the cell monolayer. Floating cell debris was removed by washing with PBS, and the medium was exchanged with serum-free medium containing various concentrations of DMSO (The negative control), allantoin(the positive control) and gentisic acid (0, 1, 5, 10, 50, and 100 μg/ml). Cells were incubated for 24 hours under the same conditions. The wound healing rate was calculated by comparing the images immediately after the scratch and 24 hours after incubation. The data was analyzed using an EVOS XL imaging system (Fisher Scientific, USA). The calculation was done by comparing the distance between wound surfaces with the 0h and 24h results. The experiments were independently repeated three times 25, 27, 31.

### Western blot

HaCaT cells (10^6^ cells) were seeded into a 90mm dish and incubated for 24hours under the same condition. The medium was exchanged with serum-free medium containing various concentrations of gentisic acid (0, 1, 5, 10, 50, and 100 μg/ml) and cells were incubated for an additional 24 hours. Equal amounts (15μg) of whole-cell lysate proteins were separated on an 8% acrylamide SDS-PAGE gel and transferred onto polyvinylidene fluoride (PVDF, Bio-Rad, USA) membrane. The membranes were blocked using 5% bovine serum albumin (BSA, GenDEPOT, Korea) and then stained with primary antibodies (p38α, p-p38, ERK1/2, JNK, p-JNK, and GAPDH from Santa Cruz Biotechnology, CA, USA; p-ERK1/2 from Cell Signaling Technology, MA, USA) overnight at 4°C. The membranes were washed three times in TBST and incubated with a secondary horseradish-peroxidase conjugated antibody (goat anti-rabbit IgG-HRP from Santa Cruz Biotechnology, CA, USA; goat anti-mouse IgG-HRP from Bio-Rad, USA) for 1 hour at room temperature. The membranes were developed using enhanced ECL (Bio-Rad, USA) on a UVITEC imaging system (UVITEC Cambridge, UK). Each lane was quantified by GAPDH 11, 25, 32.

### Statistical analysis

Results are expressed as means ± SD. Statistically significant differences were analyzed using a one-way ANOVA with Tukey's post hoc test 25.

## Results

### Gentisic acid increased keratinocyte cell proliferation

Cell proliferation assays on keratinocytes were performed using several candidate plant-originating acidic natural compounds (Table 1). Gentisic acid resulted in the highest proliferation rate (122.58%) compared to the other compounds.

**Figure 2:**
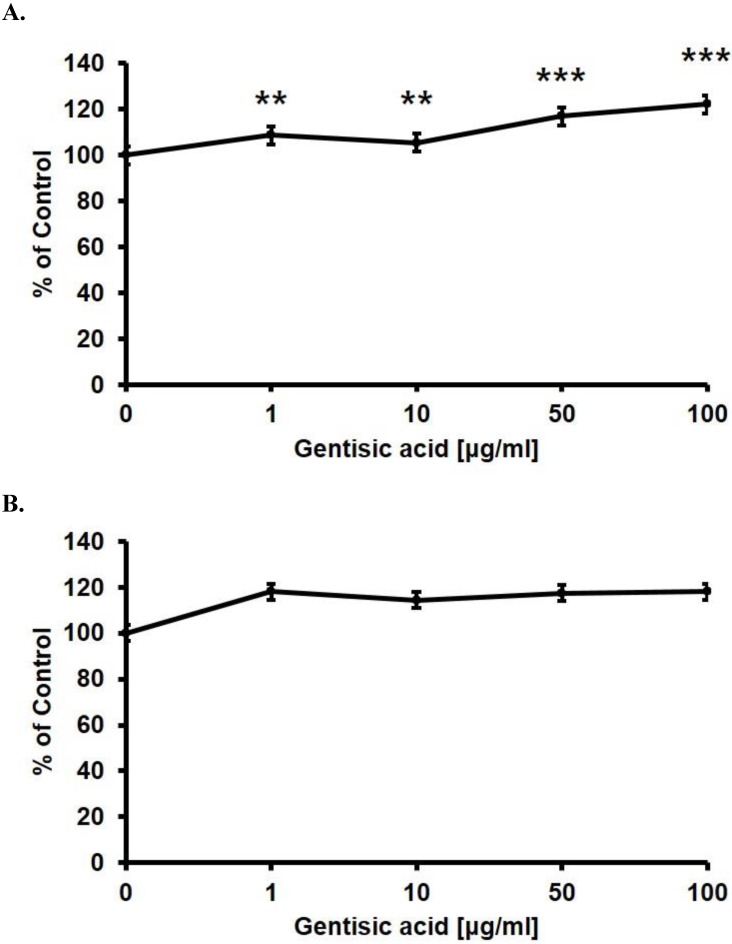
** Gentisic acid induced skin cells proliferation.** HaCaT (A) and CCD-986sk (B) cells were treated with various concentrations of gentisic acid. The cell proliferation rate was evaluated with an MTT assay. (** : P < 0.01, *** : P < 0.001)

After we chose gentisic acid as a potentially effective wound healing compound, we treated HaCaT cells with various gentisic acid concentrations in serum-free medium for 24 hours. We then evaluated the cell proliferation rate using an MTT assay (Figure 2 A). Gentisic acid dose-dependently increased the viability of HaCaT cells.

Besides, we performed the same assay on the CCD-986sk, a human dermal fibroblast cell line, to confirm whether gentisic acid is toxic to other skin cells (Figure 2 B). Gentisic acid was not toxic to both skin cell lines at the whole concentration, and cell viability of CCD-986sk cells was also increased. This result suggests that gentisic acid is not toxic to human skin cells and may improve fibroblast function during wound healing.

### Gentisic acid promoted wound healing activity

**Figure 3:**
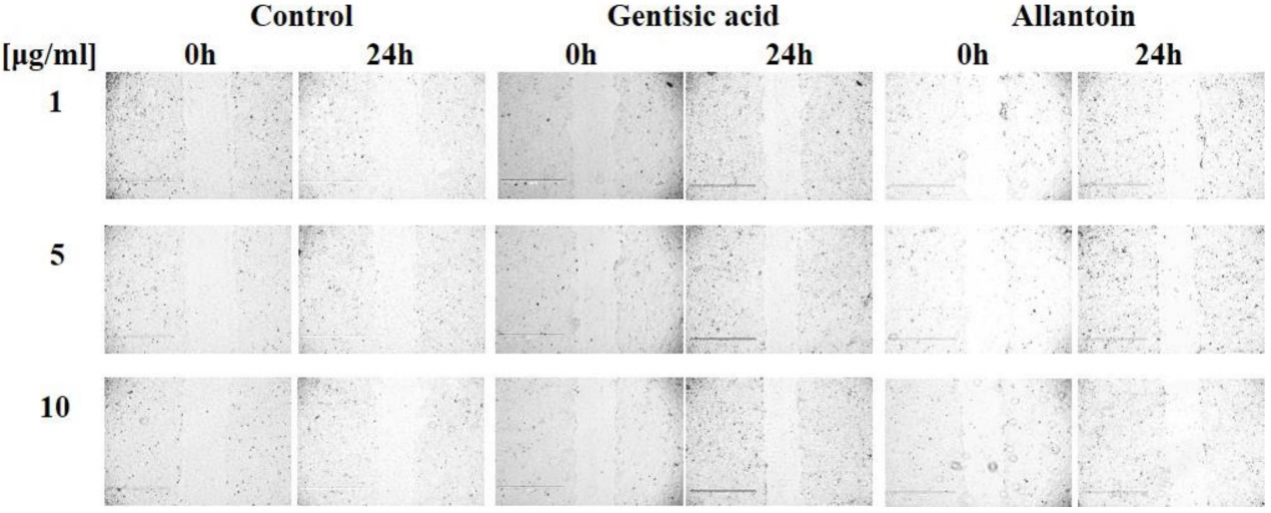
** Gentisic acid increased *in vitro* wound healing.** HaCaT cells were cultured in a 6-well plate, scratched, and treated with different concentrations of DMSO only (negative control), gentisic acid, or allantoin (positive control). The results were imaged to show scratched wound healing with various compound concentrations.

To measure the effect of gentisic acid on keratinocyte wound healing, HaCaT cells were treated with various concentrations of gentisic acid and allantoin (positive control) (Figure 3).

Gentisic acid increased wound healing of HaCaT cells by 16.81%, 15.01% and 17.98% in 1, 5, and 10 μg/ml, respectively, after 24 hours of treatment (Figure 3, Table 2).

These results showed that gentisic acid has a positive effect on keratinocyte re-epithelization by promoting proliferation and migration. Moreover, gentisic acid more potently increased cell proliferation than allantoin, which promotes cell proliferation 23.

### Gentisic acid induced ERK1/2 phosphorylation

**Figure 4:**
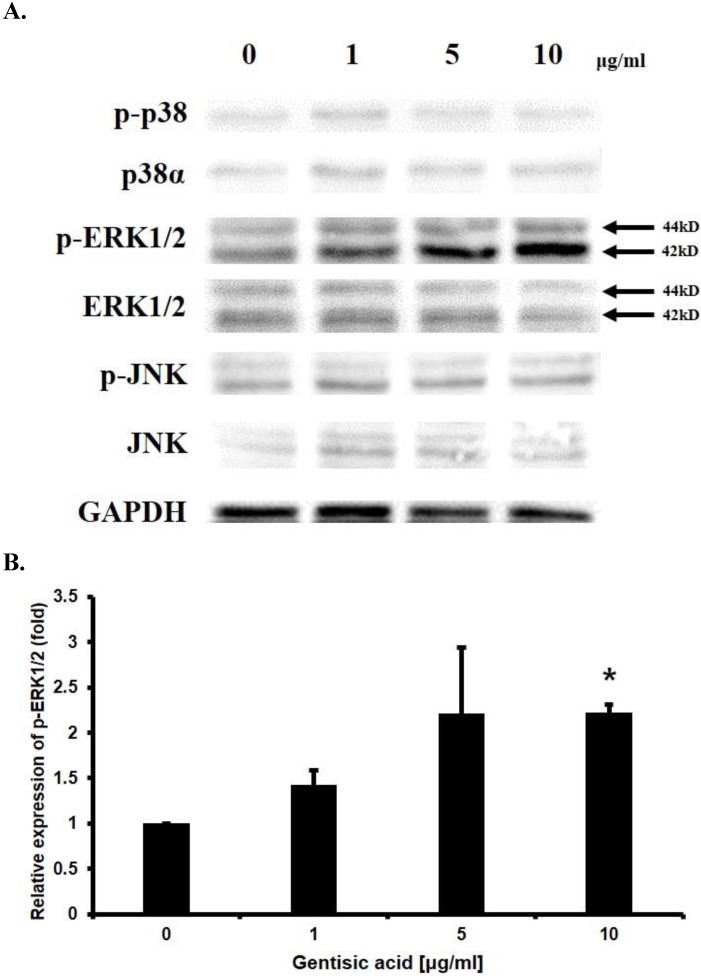
** Gentisic acid induced ERK1/2 phosphorylation in HaCaT cells.** HaCaT cells were treated with various concentration of gentisic acid and the protein extracts were used in Western blot analysis. (A) GAPDH was used as a control. p38, ERK1/2, and JNK, which in their phosphorylated forms are associated with cell proliferation, were detected. (B) p-ERK1/2 was quantified with densitometric analysis and normalized to GAPDH. (* : P < 0.05)

To determine the mechanism underlying the effect of gentisic acid on enhancing HaCaT cell proliferation, Western blotting was performed on p38α, p-p38, ERK1/2, p-ERK1/2, JNK, p-JNK which are known to be involved in cell proliferation (Figure 4A and 4B). The amount of phosphorylated ERK1/2 was increased up to 2.5 times by treatment with gentisic acid. These results indicated that gentisic acid activates the pathway associated with ERK1/2 in the MAP kinase pathway and increases the proliferation of keratinocyte.

## Discussion

Gentisic acid is a natural compound widely present in plants, and many biological studies have been conducted. The plant extract of *Alchornea cordifolia*, containing gentisic acid, has been used as a traditional African medicine 33 and it has wound healing effects 22. However, the core component of wound healing effects and its intracellular mechanism have yet to be found. In this study, we showed that gentisic acid positively affects the proliferation of the keratinocyte, HaCaT.

During epidermal wound healing, recovery of the damaged area by keratinocyte is very important 2, 3. This process is determined by the proliferation and migration of keratinocyte. In the MTT and *in vitro* wound healing assays, we demonstrated that gentisic acid had an ability to enhance keratinocyte proliferation. Proliferation and migration of fibroblasts in the proliferative phase are also important as keratinocyte 1. CCD-986sk, human skin fibroblast cell line, was not toxic to gentisic acid and its proliferation also induced by gentisic acid. In our study, the maximum value of increased proliferation by gentisic acid on keratinocytes was 122.58 %. Comparing the results with an example of other compounds, the lichen compounds, conducted for similar purposes, it seems that the efficacy of proliferation promoted is similar to that of our experiment 34. Therefore, the efficacy of gentisic acid seems to be meaningful *in vitro* measurements.

In terms of the intracellular signaling pathway, the proliferation of keratinocyte is related to the MAP kinase pathway 8. Western blot results suggested that gentisic acid induced ERK1/2 phosphorylation, one of the MAP kinases, implying an increase in HaCaT cell proliferation. In contrast, the effect of gentisic acid on p38 and JNK kinases, which are associated with proliferation, was not influenced in this result 7. The phosphorylation of ERK1/2 does not always inducing the proliferation of the cell, also induced in apoptosis stress condition and its phosphorylation can mediate the apoptosis of the cell 35. Until it is unclear how phosphorylation of ERK1/2 under various conditions promotes the expression of the exact gene. Our further study is to determine the downstream action of phosphorylated ERK1/2 after treating the compound that regulates keratinocyte proliferation by regulating the MAP kinase pathways such as gentisic acid.

In conclusion, *in vitro* condition, gentisic acid was not toxic to the keratinocyte and fibroblast and effective for skin wound healing by promoting keratinocyte proliferation. Gentisic acid could be considered as a lead compound for wound healing and skin proliferation.

## Figures and Tables

**Table 1:** Effect of plant extract compounds on cell proliferation (% of control).

Compounds	Proliferation rate (100 μg/ml)
Esculentic acid	0.92±0.39
Actinidic acid	69.36±5.72
Pimaric acid	24.39±2.71
Corosolic acid	16.39±8.71
Caffeic acid	70.21±11.95
Loganic acid	83.12±13.23
Gallic acid	19.92±0.47
Salvianolic acid A	55.19±0.81
cis-8,11,14-Eicosatrienoic acid	9.88±0.34
Linoleic acid	8.87±0.10
γ-Linolenic acid	10.05±0.78
Gentisic acid	122.13 ± 3.30

**Table 2:** Results of an *in vitro* wound healing assay after a 24 hour gentisic acid treatment.

Concentration of treatment	Wound healing rate from 0 hours (%)		
	Control	Gentisic acid	Allantoin
1 μg/ml	7.20 ± 4.27	16.81 ± 5.07	11.17 ± 2.38
5 μg/ml	9.13 ± 1.94	15.01 ± 3.80	10.07 ± 2.26
10 μg/ml	10.38 ± 0.19	17.98 ± 6.67	10.08 ± 2.42
